# Combination of a high-fat diet with sweetened condensed milk exacerbates inflammation and insulin resistance induced by each separately in mice

**DOI:** 10.1038/s41598-017-04308-1

**Published:** 2017-06-21

**Authors:** Laureane Nunes Masi, Amanda Roque Martins, Amanda Rabello Crisma, Cátia Lira do Amaral, Mariana Rodrigues Davanso, Tamires Duarte Afonso Serdan, Roberta Dourado Cavalcante da Cunha de Sá, Maysa Mariana Cruz, Maria Isabel Cardoso Alonso-Vale, Rosângela Pavan Torres, Jorge Mancini-Filho, Joice Naiara Bertaglia Pereira, Marta Maria da Silva Righetti, Edson Aparecido Liberti, Sandro Massao Hirabara, Rui Curi

**Affiliations:** 1 Interdisciplinary Post-graduate Program in Health Sciences, Cruzeiro of Sul University, Sao Paulo, Brazil; 2 Department of Physiology and Biophysics, Institute of Biomedical Sciences, University of Sao Paulo, Sao Paulo, Brazil; 3 Campus of Exact Sciences and Technology, State University of Goias, Anapolis, Brazil; 4 Department of Biological Sciences, Institute of Biomedical Sciences, Federal University of Sao Paulo, Sao Paulo, Brazil; 5 Department of Food and Experimental Nutrition, Faculty of Pharmaceutical Sciences, University of Sao Paulo, Sao Paulo, Brazil; 6 Department of Anatomy, Institute of Biomedical Sciences, University of Sao Paulo, Sao Paulo, Brazil

## Abstract

Obesogenic diets increase body weight and cause insulin resistance (IR), however, the association of these changes with the main macronutrient in the diet remains to be elucidated. Male C57BL/6 mice were fed with: control (CD), CD and sweetened condensed milk (HS), high-fat (HF), and HF and condensed milk (HSHF). After 2 months, increased body weight, glucose intolerance, adipocyte size and cholesterol levels were observed. As compared with CD, HS ingested the same amount of calories whereas HF and HSHF ingested less. HS had increased plasma AST activity and liver type I collagen. HF caused mild liver steatosis and hepatocellular damage. HF and HSHF increased LDL-cholesterol, hepatocyte and adipocyte hypertrophy, TNF-α by macrophages and decreased lipogenesis and adiponectin in adipose tissue (AT). HSHF exacerbated these effects, increasing IR, lipolysis, mRNA expression of F4/80 and leptin in AT, Tlr-4 in soleus muscle and IL-6, IL-1β, VCAM-1, and ICAM-1 protein in AT. The three obesogenic diets induced obesity and metabolic dysfunction. HS was more proinflammatory than the HF and induced hepatic fibrosis. The HF was more detrimental in terms of insulin sensitivity, and it caused liver steatosis. The combination HSHF exacerbated the effects of each separately on insulin resistance and AT inflammatory state.

## Introduction

Obesity is an independent high risk factor for metabolic diseases such as type 2-diabetes and non-alcoholic fatty liver disease. A high intake of fat or sugar induces obesity and associated co-morbidities, such as insulin resistance, hyperglycaemia and dyslipidaemia in humans and experimental animals^1, 2^. Mice fed high-energy food are used as an experimental model to investigate the mechanisms associated with dysfunction in metabolism^3–5^. Several investigators have combined macronutrients fat and sugar (fructose) to induce the main features of metabolic disorders observed in humans^6, 7^. The different compositions of energy-dense foods can increase weight and lead to insulin resistance with varying intensities. Maioli *et al*.^6^ reported that in C57BL/6 mice, compared with other obesogenic diets that have been reported to induce obesity or metabolic disorders, a diet rich in sucrose and lipids induces a more prominent body weight gain and increase in fasting blood glucose levels. The authors reported a reduced frequency of regulatory T cells as well as decreased levels of anti-inflammatory cytokines (TGF-β and IL-10) in adipose tissue^6^. Obese patients regularly consume a diet rich in sugar and fat. However, whether fat or sugar has detrimental effects on metabolism remains to be investigated. In this study, we examined the effects of a high-fat diet, a high-sugar diet and a combination of high-fat and high-sugar in the diet on inflammation intensity and insulin resistance in C57BL/6 mice. Mice fed with high-sugar diet had free access to a sweetened condensed milk containing 68% in energy as carbohydrates.

## Results

Three groups of mice were fed obesogenic diets differing in macronutrient composition and total calories. The CD mice served as the baseline. The high quantity of fat in the diet decreased the calorie ingestion by 22% in the HF group and by 14% in the HSHF group. There was no difference in the calorie intake in the HS and CD groups (Table 1). Considering the total calorie ingestion and the energy composition of the diet, the CD and HS groups ingested carbohydrate as the main macronutrient source, 536.9 kcal and 541.4 kcal per week, respectively. The HF group ingested fat as the main macronutrient source (325.6 kcal per week), and the HSHF group ingested the combination of fat (297.5 kcal per week) and carbohydrate (228.4 kcal per week). The consumption of the HS and HF as compared to the CD led to a marked increase in body weight (increases by 4.8-fold and 5.3-fold, respectively) during the experimental period. The HSHF led to a more pronounced increase (by 9.3-fold) (Table 1).

**Table 1:** Body weight, food intake and serum metabolites levels in mice fed either the control diet or obesogenic diets (high-sugar (HS), high-fat (HF) and high-sugar/high-fat (HSHF)) for eight weeks.

	CD	HS	HF	HSHF
Initial body weight (g)	26.0 ± 0.67	25.0 ± 0.55	27.0 ± 0.70	25.0 ± 0.57
Body weight gain (g)	1.9 ± 0.42	9.9 ± 0.98^a^	8.9 ± 1.10^a^	17.5 ± 1.41^a,b,c^
Food ingestion (g/week)	177.2 ± 7.13	130.2 ± 4.86^a^	102.7 ± 1.17^a,b^	82.1 ± 1.66^a,b,c^
Condensed milk intake (g/week)	—	74.8 ± 4.67	—	51.8 ± 6.57^b^
Caloric ingestion (kcal/week)*	673.7 ± 27.01	738.0 ± 15.99	548.5 ± 6.25^a,b^	606.8 ± 24.66^a,b,c^
Visceral adipose tissue (g)	0.64 ± 0.19	1.3 ± 0.24^a^	1.7 ± 0.41^a^	2.0 ± 0.26^a,b^
Liver weight (g)	1.2 ± 0.04	1.8 ± 0.15^a^	1.8 ± 0.13^a^	2.0 ± 0.12^a^
Total cholesterol (mg/dL)	137.0 ± 7.42	186.0 ± 5.84^a^	201.0 ± 7.82^a^	207.0 ± 8.52^a^
LDL-cholesterol (mg/dL)	85.0 ± 7.30	100.0 ± 4.27	140.0 ± 9.37^a,b^	114.0 ± 6.72^a^
Triacylglycerol (mg/dL)	62.0 ± 5.90	56.0 ± 3.41	66.0 ± 4.25	72.0 ± 3.29
AST (U/L)	13.8 ± 1.25	31.2 ± 4.23^a^	18.9 ± 2.39	25.1 ± 4.32
Fasted** serum glucose (mg/dL)	163.0 ± 7.84	190.0 ± 6.83	240.0± 13.64^a,b^	214.0 ± 13.49^a^
Fasted** serum insulin (ng/mL)	0.3 ± 0.03	1.1 ± 0.12	1.2 ± 0.25	2.4 ± 0.45^a,b,c^
Glucose intolerance (AUC)	8154 ± 840.6	14209 ± 1305^a^	14409 ± 1372^a^	18574 ± 1377^a^
Insulin sensitivity (kITT - %/min)	3.5 ± 0.25	3.1 ± 0.20	3.7 ± 0.28	2.2 ± 0.21^a,b,c^

*Caloric ingestion considered the food ingestion calories for CD and HF group and food ingestion plus condensed milk intake calories for HS and HSHF groups; **Fasted for 3–4 hours. CD, control diet; HS, high-sugar diet; HF, high-fat diet; HSHF, high-sugar/high-fat diet; LDL-cholesterol, low density lipoprotein cholesterol; AST, aspartate aminotransferase; AUC, area under the curve of glycaemia - values obtained from the glucose tolerance test (GTT); kITT (%/min), the constant rate for plasma glucose disappearance - values obtained from the insulin tolerance test (ITT). The results are shown as the mean ± S.E.M. (n = 12–13). The values were analysed using the one-way ANOVA and Tukey’s post-test. (a) *P* < 0.05 versus CD; (b) *P* < 0.05 versus HS; and (c) *P* < 0.05 versus HF.

**Figure 1:**
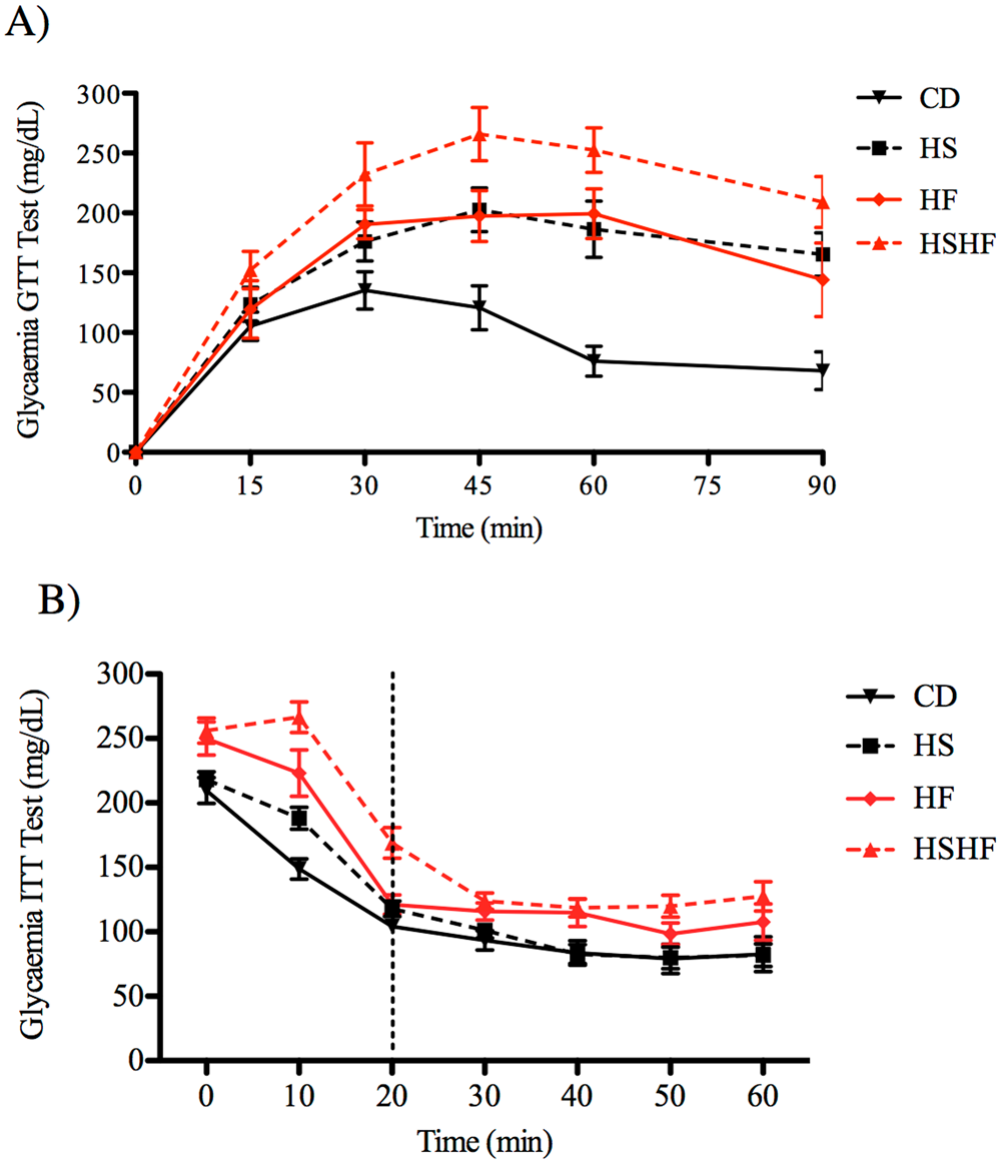
Variations in blood glucose levels during the (**A**) glucose tolerance test (GTT) and (**B**) insulin tolerance test (ITT) in mice fed the control diet (CD) or obesogenic diets (high-sugar (HS), high-fat (HF) or high-sugar/high-fat diet (HSHF) for 8 weeks. The results are expressed as the mean ± S.E.M. (n = 4–7/group).

Compared to the CD, the obesogenic diets (HS, HF and HSHF) induced a consistent increase in visceral adipose tissue (mesenteric, epididymal and perirenal) weight by 2-, 2.7- and 3-fold, respectively, liver wet weight by 50%, 50% and 67%, respectively, and glucose intolerance intensity (as indicated by the area under the curve) by 70%, 80% and 130%, respectively (Fig. 1A and Table 1).

A high quantity of fat in the diet (HF and HSHF) induced an increase in serum LDL-cholesterol levels by 65% and 34%, respectively, and in glycaemia by 47% and 31%, respectively, compared with the CD animals (Table 1). A high quantity of sugar in the diet (HS) increased AST activity by 2.3-fold compared to the CD (Table 1). The combination of high sugar and high fat (HSHF group) decreased insulin sensitivity (as indicated by the kITT) by 29% and 40%, respectively, increased body weight by 77% and 97%, respectively, and 6-hour fasting serum insulin levels by 2-fold compared with the HS and HF groups, respectively (Fig. 1B and Table 1).

**Figure 2:**
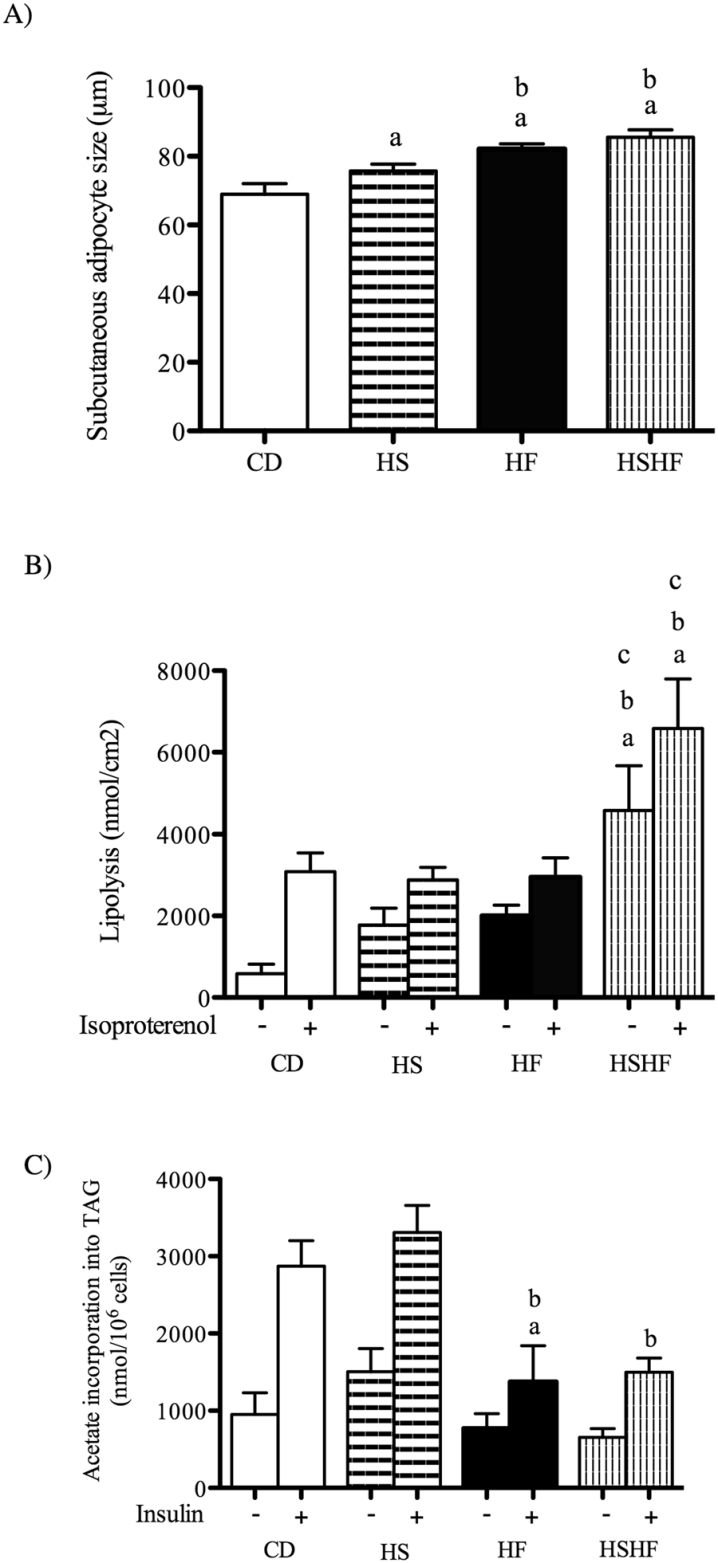
Inguinal adipocyte size (**A**), lipolysis (**B**) and lipogenesis (**C**) rates in mice fed the control diet (CD) or obesogenic diets (high-sugar (HS), high-fat (HF) and high-sugar/high-fat diet (HSHF) for 8 weeks. The results are expressed as the mean ± S.E.M. (n = 4–7/group). (a) *P* < 0.05 compared with CD, (b) *P* < 0.05 compared with HS, and (c) *P* < 0.05 compared with HF, using one-way ANOVA and Tukey’s post-test.

Subcutaneous adipocyte size was increased by the three obesogenic diets (HS by 10%; HF by 19%; HSHF by 24%) compared to CD diet (Fig. 2A). The HF and HSHF groups increased adipocyte size by 8.5% and 13%, respectively, compared to the HS group (Fig. 2A).

The combination of sugar and fat (HSHF) led to a marked increase in body weight. This was the only group that showed increased lipolysis in incubated subcutaneous adipocytes tissue in both un-stimulated (by 7.8-fold compared with CD; by 2.6-fold compared with HS and by 2.3-fold compared with HF) and isoproterenol-stimulated conditions (by 2-fold compared with all the others) (Fig. 2B). The high fat content in the diet (in the HF and HSHF groups) reduced lipogenesis in insulin-stimulated subcutaneous adipocytes by 2- and 2.4-fold, respectively, compared with the HS and HF mice, whereas there was a two-fold decrease compared with the CD mice (Fig. 2C).

**Figure 3:**
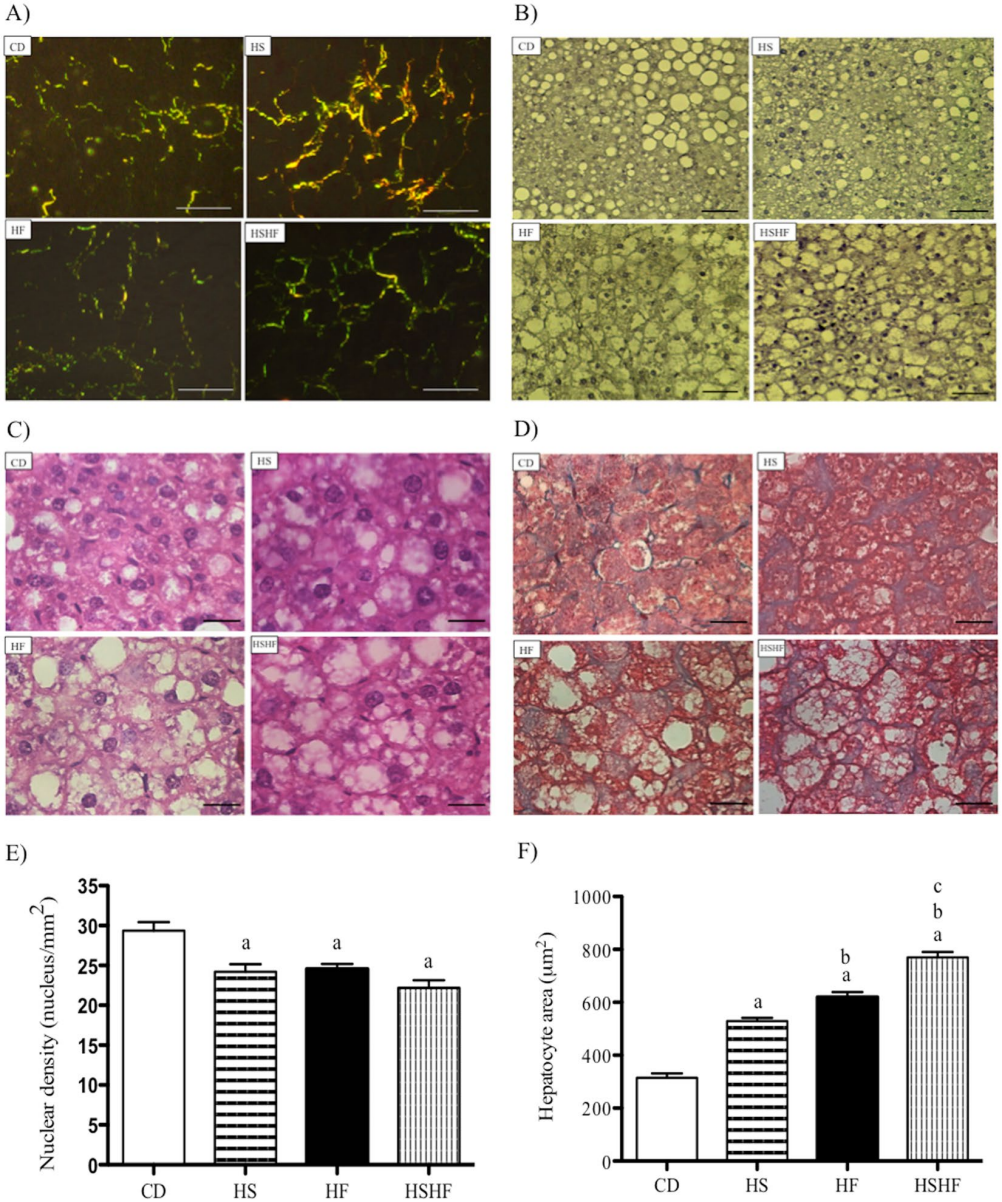
Photomicrographs illustrating the liver morphology of mice fed the control or obesogenic diets (high-sugar (HS), high-fat (HF) or high-sugar/high-fat diet (HSHF) for 8 weeks. (**A**) Picrosirius red staining; bar = 20 μm; (**B**) Sudan black staining; bar = 10 μm; (**C**) haematoxylin and eosin stain; bar = 20 μm; (**D**) azo carmine; bar = 20 μm; (**E**) nuclear density (nuclei/mm^2^) using 5 random fields/3 sections/animal; and (**F**) area of hepatocytes (μm^2^) measured in 45 cells per animal. The results are expressed as the mean ± S.E.M. (n = 4–7/group). (a) *P* < 0.05 compared to CD, (b) *P* < 0.05 compared to HS, and (c) *P* < 0.05 compared to HF, using one-way ANOVA and Tukey’s post-test.

The high-sugar diet (HS group) increased type I collagen deposition in the liver as demonstrated by the qualitative analyses, using picrosirius red staining, compared with the others (Fig. 3A). The livers from high-fat-diet-fed mice had large lipid droplets, as indicated by Sudan staining (Fig. 3B). The obesogenic diets also induced cell death, as indicated by the decreased density of nuclear staining with haematoxylin and eosin (HS by 18%; HF by 16.5% and HSHF by 24.5%, compared with CD) (Fig. 3C,E) and led to an increase in the hepatocyte area (HS by 1.7-fold; HF by 2-fold and HSHF by 2.5-fold, compared with CD) (Fig. 3D,F).

The supply of condensed milk (HS group) increased the mRNA expression of *leptin* in eAT (by 3.3-fold) and *type I collagen* (by 2-fold) in the liver and decreased *leptin* mRNA expression (by 3.6-fold) in the soleus muscle (Table 2) compared with the CD group. The HF mice showed increased mRNA expression of *leptin* (by 3.5-fold) and decreased expression of *adiponectin* (by 2.8-fold) in eAT compared with the CD group (Table 2). The combination of sugar and fat (HSHF group) increased the mRNA expression of *F4*/*80* (by 5.7-fold) and decreased expression of *adiponectin* (by 2.8-fold) in eAT and increased *Tlr*-*4* mRNA expression (by 1.6-fold) in the soleus muscle compared to the CD group (Table 2).

**Table 2:** mRNA expression of inflammatory genes in insulin-responsive tissues: adipose tissue, liver and skeletal muscle from mice fed the control diet (2) or obesogenic diets: high-sugar (HS), high-fat (HF) and high-sugar/high-fat diet (HSHF) for eight weeks.

Tissue	mRNA	CD	HS	HF	HSHF
eAT	*F4*/*80*	1 ± 0.09	2.5 ± 0.36	4.5 ± 0.81	5.7 ± 1.42^a^
	*Adiponectin*	1.4 ± 0.21	1.1 ± 0.28	0.5 ± 0.10^a^	0.5 ± 0.18^a^
	*Leptin*	1.2 ± 0.15	3.9 ± 0.40^a^	3.0 ± 0.37^a^	2.4 ± 0.35^b^
Liver	*Collagen*	1.1 ± 0.23	2.2 ± 0.37^a^	1.3 ± 0.28	1.6 ± 0.17
	*Tnf*-*α*	1.5 ± 0.41	2.9 ± 0.87^c^	0.7 ± 0.14	1.1 ± 0.27
Soleus Muscle	*Leptin*	1.1 ± 0.25	0.3 ± 0.07^a,c^	1.4 ± 0.17	1.1 ± 0.26
	*Tlr4*	1.0 ± 0.11	1.3 ± 0.13	1 ± 0.13	1.6 ± 0.09^a,c^

mRNA gene expression in the epididymal adipose tissue (n = 6–7/group; Rplp0 as housekeeping gene), liver (n = 10–12/group; 18 S as housekeeping gene) and soleus muscle (n = 6–7/group; 18 S as housekeeping gene). The results are expressed as the mean ± S.E.M. (a) *P* < 0.05 compared with CD, (b) *P* < 0.05 compared with HC, and (c) *P* < 0.05 compared with HF using one-way ANOVA and Tukey’s post-test. CD, control diet; HS, high-sugar diet; HF, high-fat diet; HSHF, high-sugar/high-fat diet; *Tnf* –tumour necrosis factor; *Tlr4*, Toll-like receptor 4.

**Figure 4:**
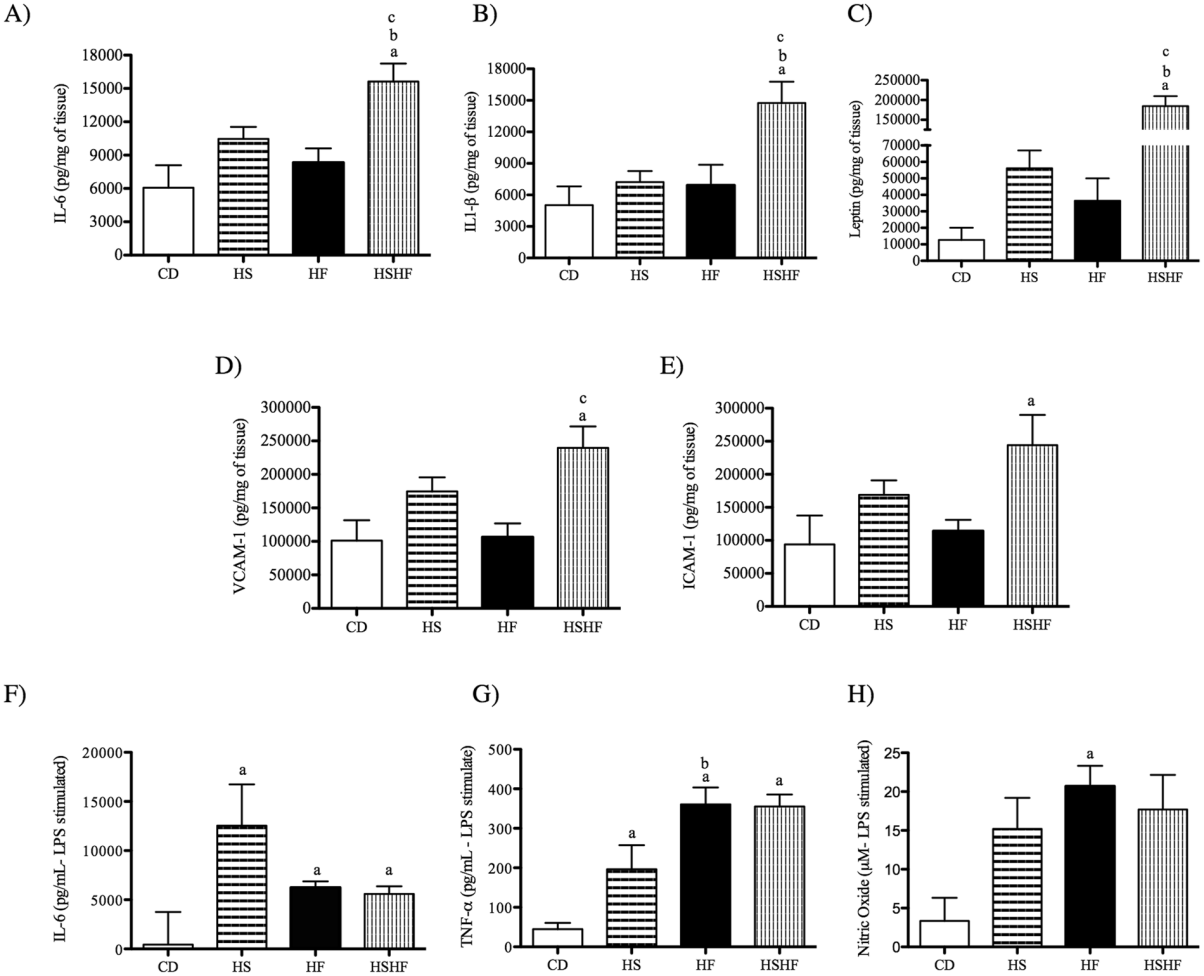
Content of IL-6 (**A**), IL-1β (**B**), leptin (**C**), VCAM-1 (**D**) and ICAM-1 (**E**) by epididymal adipose tissue and of IL-6 (**F**), TNF-α (**G**) and nitric oxide (**H**) by LPS-stimulated peritoneal macrophages (1 × 10^6^ cells) from C57BL/6 mice fed the control diet (CD) or obesogenic diets (high-sugar (HS), high-fat (HF) or high-sugar/high-fat diet (HSHF) for eight weeks. The results are expressed as the mean ± S.E.M. (n = 4–7/group). (a) *P* < 0.05 compared with CD, (b) *P* < 0.05 compared with HS, and (c) *P* < 0.05 compared with HF, using the one-way ANOVA and Tukey’s post-test. VCAM, vascular cell adhesion protein 1; ICAM, intercellular cell adhesion molecular 1; IL, interleukin; LPS, lipopolysaccharide from *E*. *coli* 055:B5 (2.5 μg/mL for 24 h).

Only the combination high-sugar and high-fat diet (HSHF group) induced a significant increase in proinflammatory protein production in epididymal adipose tissue compared with the other three groups: IL-6 (by 2.6-fold compared with CD; by 1.5-fold compared with HS; by 1.9-fold compared with HF), IL1-β (by 2.9-fold compared with CD; by 2-fold compared with HS and HF), leptin (by 14.5-fold compared with CD; by 3-fold compared with HS; by 5-fold compared with HF), VCAM-1 (by 2.4-fold compared with CD; by 2.3-fold compared with HF) and ICAM-1 (by 2.6-fold compared with CD) (Fig. 4A–E, respectively).

The LPS-stimulated peritoneal macrophages demonstrated a striking increase (by 28-fold) in IL-6 production in mice fed the HS diet compared to the CD diet (Fig. 4F). The HF and HSHF diets induced a 8-fold increase in TNF-α production compared with the CD diet (Fig. 4G), and only the HF diet increased nitric oxide production (by 6.2-fold) compared with peritoneal macrophages from the CD mice (Fig. 4H).

## Discussion

High-calorie diets induce obesity and associated co-morbidities such as insulin resistance, non-alcoholic fatty liver disease (NAFLD) and chronic low-grade inflammation^8–11^. These diets present high contents of sugar, fat or both. The aim of this study was to investigate whether a marked increase in one of the macronutrients, fat or sugar, or both, could affect the consequences of obesity on inflammation and insulin resistance. The mice were fed one of three obesogenic diet regimes: control diet with free access to sweetened condensed milk, high-fat diet, or high-fat diet with free access to sweetened condensed milk. The main macronutrient present in the sweetened condensed milk used and that is usually commercially available at the market is carbohydrate (~53% sucrose and ~15% lactose)^12^. The three obesogenic diets induced body weight gain, glucose intolerance and increased visceral fat depots and liver wet weight, adipocyte size and serum total cholesterol levels compared to the control diet. These changes were more pronounced in the high-sugar/high-fat diet group and characterize a metabolic syndrome condition.

High-fat- and high-sugar/high-fat-fed mice ingested fewer calories whereas the high-sugar diet group ingested an equal calorie quantity as the control group. The consensus is that higher caloric intake induces higher body weight gain irrespective of the source^13^. The overconsumption of rapidly absorbable carbohydrates and palatable food such as condensed milk causes a rapid elevation of serum insulin levels, and food craving then occurs^14, 15^. Hall^16^ described the carbohydrate-insulin model in which diets with a high proportion of carbohydrates elevate insulin secretion, thereby suppressing the release of fatty acids from the adipose tissue into the circulation and direct the circulating fatty acids towards the adipose tissue storage and away from oxidation by metabolically active tissues, such as heart, muscle and liver. This altered fuel availability and distribution may lead to a state of cellular ‘internal starvation’, decreased energy expenditure and increased hunger^17^.

Fat is the highest caloric component in the diet (9 kcal/g compared with 4 kcal/g for carbohydrate)^18^ but it suppresses appetite^19^. Recently, Olsen *et al*.^20^ reported that old male C57BL/6 J mice fed a high-fat diet (60% fat) for five weeks had increased body weight but plateaued 6 weeks after commencing the high-fat diet feeding (at 11 weeks of age). The authors also reported no difference in calorie intake between the HF and control mice neither during the light phase nor during the dark phase. The energy expenditure was measured, and the basal metabolic rate remained unchanged in obese mice compared with balanced chow-fed controls. The active metabolic rate seemed to be significantly reduced in obese mice. Additionally, diet-induced thermogenesis studies reported that lower energy was dissipated as heat after digestion of fat (~7%) compared to sucrose (~11.4%)^21^.

Free access to condensed milk induced an increase in serum AST activity and type I collagen deposition in the liver, as demonstrated by morphological analysis and confirmed by mRNA expression. Condensed milk has approximately 53% sucrose, a disaccharide that contains glucose and fructose. In humans, increased fructose consumption is associated with an increased severity of hepatic steatosis and fibrosis^22, 23^. The ingestion of sucrose containing fructose most likely accelerates the development of liver fibrogenesis^24, 25^. High sugar intake also promotes inflammation, as demonstrated by augmented peritoneal macrophage IL-6 production and *Tnf*-*α* mRNA expression in the liver. Some authors have also described a link between leptin resistance and non-alcoholic steatohepatitis (NASH) development^26–28^.

NAFLD refers to a spectrum of liver diseases, including non-alcoholic fatty liver, which is characterized by steatosis with no or minor inflammation, and NASH, which is associated with inflammation and ballooning with or without fibrosis^29^, and it may progress to liver cirrhosis and hepatocellular carcinoma^30, 31^. As reported in this study and in work by others, the livers from mice fed a high-fat diet lacked fibrosis and showed mild steatosis and focal hepatocellular necrosis and apoptosis; their features were compatible with the progression of steatosis in NASH^32^. The increased peritoneal macrophage production of nitric oxide induced by a high-fat diet described in this study may contribute to NASH disease development. NO is involved in NASH progression, including mitochondrial dysfunction^33^ and biogenesis^34^.

Substituting a high-fat diet with a high-carbohydrate diet is associated with a decrease in LDL particle size and an increase in LDL density, which contribute to atherogenic dyslipidaemia^35, 36^. The high-fat diet increased the plasma levels of LDL-cholesterol, glycaemia, TNF-α production by peritoneal macrophages, led to hepatocyte and adipocyte hypertrophy, and decreased lipogenesis and adiponectin content in adipose tissue, regardless of the inclusion of condensed milk. This observation indicates an important fat-diet-induced metabolic dysfunction. Adipocyte and hepatocyte hypertrophy and inflammatory responses are associated with the development of insulin resistance and consequently increased glycaemia^37^. Adiponectin increases fatty acid β-oxidation^38^ and it is often lower in the plasma of obese subjects^39^. Liu *et al*.^40^ reported that peritoneal macrophages from diet-induced obese mice exhibit impaired autophagy with increased TNF-α production. Lipogenesis is stimulated by high food intake to promote triglyceride storage^41^. Brunengraber *et al*.^42^ reported reduced epididymal fat pad lipogenesis in mice consuming a lard-based high-fat diet compared with a high-carbohydrate diet.

**Figure 5:**
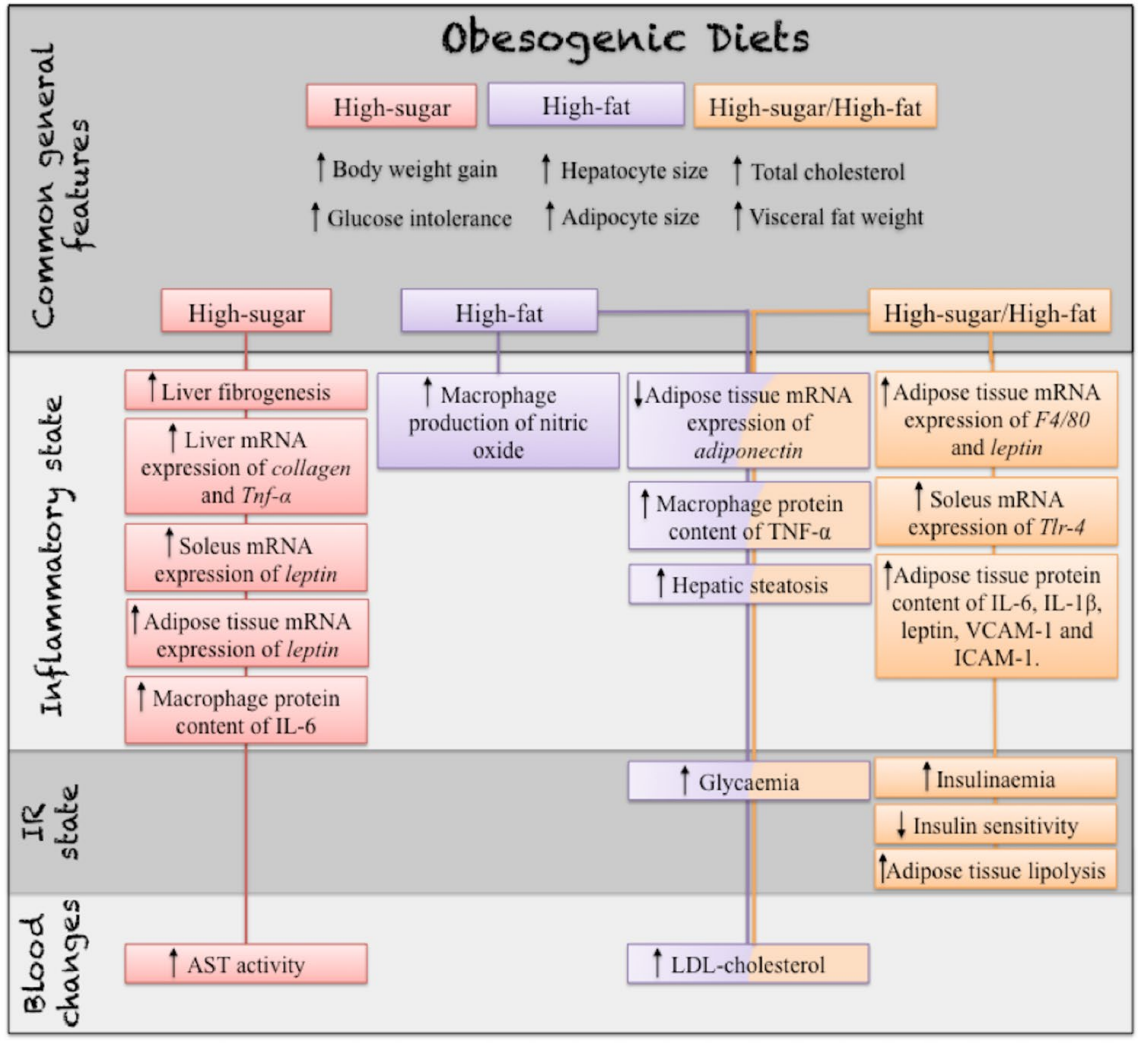
Summary of the effects of the three obesogenic diets, high-sugar (HS), high-fat (HF) and high-sugar/high-fat (HSHF), given to the mice for eight weeks. Up arrows indicate an increase, and down arrows indicate a decrease. The descriptions of the effects descriptions were subdivided into common general features, inflammatory state, insulin resistance (IR) state and changes in blood. AST, aspartate aminotransferase; VCAM, vascular cell adhesion protein 1; ICAM, intercellular cell adhesion molecular 1; IL, interleukin; TNF, tumour necrosis alpha; Tlr-4, toll-like receptor 4; IR, insulin resistance.

The high-sugar/high-fat diet intensified the effects induced by the two obesogenic diets separately, resulting in increased fasting serum insulin levels, insulin resistance, lipolysis, mRNA expression of *F4*/*80* and *leptin* in adipose tissue, mRNA expression of *Tlr*-*4* in soleus muscle and protein content of IL-6, IL-1β, leptin, VCAM-1 and ICAM-1 in adipose tissue (Fig. 5). The HSHF group ingested as many calories as the control group. Obesity and its associated co-morbidities were aggravated by the combination of both. Maioli *et al*.^6^ reported that mice fed a high-sugar and butter diet for 11 weeks exhibited, as demonstrated in our study, changes compatible with metabolic syndrome and more intense inflammation compared with mice fed chow, an AIN93G diet, a high-sugar or a high-fat diet.

Animal models of nutrition have been developed to mimic the alimentary habits that culminate in obesity and related disorders as NAFLD in humans. The sweetened condensed milk was more inflammatory than the high-fat diet and induced hepatic fibrosis. The high-fat diet was more detrimental for peripheral insulin sensitivity, and it caused liver steatosis. The ingestion of the combination high-sugar and high-fat diet intensified all changes induced by the high-sugar and high-fat diets individually. A summary of the findings reported is in the Fig. 5.

## Material and Methods

### Ethical approval

The animal studies were performed according to protocols approved by the Animal Care Committee of the Institute of Biomedical Sciences, University of São Paulo, Sao Paulo, Brazil (125/10/CEUA). All experiments were performed in accordance with relevant guidelines and regulations.

### Animals

Male C57BL/6 mice (12 weeks old) were housed in a room with a light-dark cycle of 12–12 h and temperature of 23 ± 2 °C. The mice were divided into two groups and were fed a control diet (CD) (energy composition of 76% carbohydrates, 9% fat, 15% proteins; 3.8 Kcal/g) or a high-fat diet (HF) (energy composition of 26% carbohydrates, 59% fat, 15% proteins; 5.3 Kcal/g) for 8 weeks. In both diets, the main source of fat was lard, and the main source of carbohydrate was corn starch. A similar protocol was applied in our previous studies^43–46^. Approximately 50% of the CD and HF mice, concomitantly with the diet, received a separate bowl of sweetened condensed milk (energy composition of 68% carbohydrates, 23% fat, 9% protein; 3.25 Kcal/g) (Italac, Sao Paulo, SP, Brazil) supplemented with a vitamin and mineral mix (Rhoster, Sao Paulo, SP, Brazil) to generate two other groups: high-sugar (HS) and high-fat and high-sugar (HFHS)^47^. Condensed milk, water and both diets (CD and HF) were provided *ad libitum*. The mice were weighed once a week. Food and condensed milk intake was measured and re-issued every 2 days. Food intake ([food offered (g) − food remaining (g)]), condensed milk and calorie intake ([food intake × kcal/g of diet] + [condensed milk intake × kcal/g of condensed milk]) were calculated for each group each week in a cage consisting of 6 mice. After 8 weeks of the obesogenic diets (20 weeks old), the mice were fasted for 2–4 hours and then were killed using carbon dioxide.

### Blood measurements, glucose and insulin tolerance tests

Blood measurements, glucose tolerance tests (GTTs) and insulin tolerance tests (ITTs) were performed as described in our previous study^46^. The rate constant for the ITT (kITT) was calculated using the formula kITT (%/min) = 0.693/t½, where t½ is calculated from the slope of the plasma glucose concentration during the period from 0 to 20 minutes after insulin injection, using least squares analysis; the decline in plasma glucose concentration during this period was linear. Total cholesterol^48^, triacylglycerol^49^ and the activity of aspartate aminotransferase-AST^50^ were evaluated using colorimetric assays (Labtest Diagnostics, Lagoa Santa, MG, Brazil), and insulin was determined using ELISAs (Millipore kit, St. Charles, MO). LDL-cholesterol was calculated using the Friedewald equation^51^.

### Adipocyte isolation and adipose tissue metabolism

Subcutaneous adipocyte isolation was performed as previously described^52^ with slight modifications^53^. A small number of adipocytes were photographed using an optical microscope (×100 magnification) and a microscope camera (Moticam 1000; Motic, Richmond, British Columbia, Canada), and the mean adipocyte diameter was assessed by measuring 50 cells using Motic-Images Plus 2.0 software. Lipolysis and the incorporation of [1-^14^C]-acetate into fatty acids were assessed in subcutaneous adipocytes isolated as described in previous studies^44, 53^.

### Measurement of inflammatory markers

The inflammatory parameters were measured in the epididymal adipose tissue (eAT) [interleukin (IL)-6, IL-1β, leptin, vascular adhesion molecule-1 (VCAM-1) and intercellular adhesion molecule 1 (ICAM-1)] and peritoneal macrophages (IL-6 and tumour necrosis factor α - TNF-α) using ELISAs (DuoSet kits, R&D System, MN, USA). The values obtained for epidydimal adipose tissue were normalized to total protein content by applying the method of Bradford^54^. For epididymal adipose tissue, the total tissue weight was also used for normalization. The production of nitric oxide (NO) by peritoneal macrophages was determined using the method of Griess^55^ described in our previous study^47^.

### Gene expression analysis

The expression levels of genes involved in inflammation in eAT, liver and soleus muscle were evaluated using real-time PCR (polymerase chain reaction) as previously reported by our group^43^. The expression of *Rplp0* was used as internal control for eAT and the expression of 18S for liver and soleus muscle. Reference genes were defined in preliminary assays that indicated unaffected expression levels in the experimental conditions herein used^56, 57^. The primer sequences were: *F4*/*80*, NM_010130.4, sense CCTGAACATGCAACCTGCCAC, antisense GGGCAT GAGCAGBCTGTAGGATC, *Adiponectin*, NM_009605.4, sense TCTTAATCCTGCCCAGTCATGC, antisense TCCAACATCTCCTGTCTCACCC, *Leptin*, NM_008493.3, sense TCACACACGCAGTCGGTATCC, antisense ATGGAGGAGGTCTCGGAGATT, *Collagen*, NM_009931.2, sense CTCTATGTCCAAGGCAACGAG, antisense TCACAAACCGCACACCTG, *TNF*-*α*, NM_001278601.1, sense TCTTCTCATTCCTGCTTGTGGC, antisense CACTTGGTGGTTTGCTACGAC G, *Tlr4*, NM_021297.3, sense TTCAGAACTTCAGTGGCTGG, antisense TGTTAGTCCAGAGAAACTTCCTG, *Rplp0*, NM_007475.5, sense CCACTTACTGAAAAGGTCAAGGC, antisense TGGTTGCTTTGGCGGGATTA, *18S*, NM_030720.1, sense CGCTACACTGACTGGCTCAG, and antisense CAGGGACTTAATCAACGCAAG.

### Histomorphometric analysis of the liver

Fragments of the right lobe liver were collected, fixed in 10% paraformaldehyde for 24 hours and washed in distilled water for 6 hours for light microscopy. After the liver fragments were fixed, the material was dehydrated in ascending alcohol series, diaphanized in xylene and embedded in paraffin. Semi-serial histological cross-sections of 5 μm in thickness were stained with picrosirius red^58^ under polarized light to detect types I and III collagen fibres and with Sudan black for lipid detection. Images of hepatocytes (area and density) were captured using a camera (AxioCam) coupled to a trinocular microscope (Zeiss, Oberkochen, Germany) and were analysed using the image analysis software Axio Vision 4.3. To determine the area of hepatocytes (μm^2^), azo carmine stained sections were randomly measured (45 cells per animal). Nuclear density (nucleus/mm^2^) was calculated using 5 random fields/3 sections/animal stained with haematoxylin and eosin^59^.

### Statistical analysis

The results are presented as the mean ± standard error of the mean (S.E.M.). All groups were compared to each other using the one-way ANOVA and a Tukey post-test (GraphPad Prism, version 5.01). The differences were considered to be statistically significant for P < 0.05.
